# Characterizing app-based telemedicine for opioid use disorder treatment within the landscape of recovery-related smartphone applications

**DOI:** 10.1186/s13722-025-00635-1

**Published:** 2026-01-08

**Authors:** Lauren E. Hendy, Eileen Barrett, Cynthia Jimes, M. Justin Coffey, Marlene C. Lira

**Affiliations:** 1 Workit Health, Ann Arbor, MI 48104 USA; 2 Geisinger Commonwealth School of Medicine, Scranton, PA 18510 USA; 3 DrPH Program, Health Policy, Johns Hopkins Bloomberg School of Public Health, Baltimore, MD 21205 USA

**Keywords:** Opioid use disorder, Telemedicine, Mobile health, Health care delivery, Health services evaluation

## Abstract

A 2025 qualitative content analysis by Williamson et al. evaluated tools available through smartphone apps to support patients with recovery from opioid use disorder. Their analysis focused primarily on the role of smartphone apps as secondary, adjunct resources to addiction treatment programs. In this commentary on the analysis by Williamson et al., we discuss how apps for comprehensive telemedicine treatment for opioid use disorder are a growing segment within the mobile health offerings geared toward addiction treatment. Communities that face barriers to accessing in-person care for opioid use disorder can receive evidence-based treatment, such as medication for opioid use disorder and behavioral counseling, through rigorously tested telemedicine apps. Peer-reviewed studies from telemedicine practices have demonstrated the effectiveness and quality of virtual care models in reducing opioid use and increasing recovery. Future research on app-based telemedicine will represent an important contribution to addiction science in evaluating new therapeutic and supportive interventions for long-term recovery from opioid use disorder.

## Introduction

In response to the opioid crisis in the United States, mobile health (mHealth) interventions to reduce opioid overdose risk and treat opioid use disorder (OUD) have grown rapidly in use and content. A recent content analysis by Williamson et al. evaluated over 150 apps related to OUD recovery and highlighted the complementary role smartphone apps can play in OUD treatment delivered in brick-and-mortar clinics [1]. We appreciated the excellent review by Williamson et al., yet an area of mHealth offerings for OUD recovery that was little discussed in their work was the role of smartphone applications that deliver virtual addiction treatment while providing recovery support tools independent of brick-and-mortar clinics.

Telemedicine practices employ app-based virtual models of OUD care and have delivered evidence-based OUD treatment to thousands of patients across the United States. In these programs, smartphone apps allow patients with OUD to attend video-based visits with physicians, advanced practice providers, recovery counselors, and certified peer support specialists. Apps can offer synchronous and asynchronous support through virtual support groups, message boards, and informational resources for recovery and monitor recovery progress through virtual assessments and remote administration of urine drug screens [2]. Patients with OUD can receive recommended medications for OUD to initiate at home such as buprenorphine or naltrexone as well as treatment for co-occurring conditions such as anxiety, depression, and insomnia that may affect their recovery from opioid use.

## Growing empirical evidence on telemedicine impact

Only Boulder Care was mentioned by Williamson et al. as an empirically studied tool, yet several app-based telemedicine practices have begun to publish peer-reviewed research informing the scientific community on the impact of their clinical models of care and preliminary outcomes among patients with OUD [2–4]. The functionality of OUD telemedicine apps enables the collection of real-time data on patients describing substance use recovery and treatment patterns. Growing evidence has demonstrated the effectiveness of OUD treatment delivered via telemedicine apps including that patients receiving this care have similar or higher retention in treatment and continuous buprenorphine use as patients treated in in-person clinics [2, 3]. Lira et al. found approximately 52% of OUD patients were retained in telemedicine treatment at 6 months compared to 35 to 41% of patients treated in in-person settings in other studies [2]. Lira et al. also reported 49% of telemedicine patients were adherent to buprenorphine treatment according to urine drug testing at 6 months, while Chan et al. found the risk of buprenorphine discontinuation at 6 months was 61% lower for OUD patients in telemedicine treatment compared to in-person treatment [2, 3]. App-based telemedicine programs for OUD have also demonstrated decreases in self-reported substance use and growth in recovery capital, defined as the psychosocial behaviors and environmental resources that support addiction recovery [4]. Burke et al. found the percentage of telemedicine patients who reported zero days of opioid use in the past month increased from 50.2% to 89.5% and the mean scores for social and material protective factors for recovery on the Brief Addiction Monitor scale rose from 14.27 to 14.99 after one month of care [4]. Finally, as Williamson et al. noted, the widespread reach of smartphones could expand the reach of resources for OUD recovery. Studies from app-based telemedicine programs for OUD have shown success in treating populations who are underserved in addiction treatment due to provider shortages, transportation barriers, or stigma such as pregnant people, patients in rural areas, and people experiencing homelessness [2–5]. A chart review by Coffey et al. found, of 94 patients who initiated telemedicine treatment for OUD during a pregnancy or became pregnant after starting care, 69 received continuous OUD care through telemedicine during their pregnancies [5]. In their studies of treatment retention and substance use recovery, approximately 15% of telemedicine patients studied by Burke et al. reported being unhoused, and Lira et al. examined retention and adherence among over 1,800 patients residing in rural zip codes [2, 4].

## Expanding the scope of future research

There are particular opportunities for future research to validate the role and impact of smartphone apps for telemedicine treatment of OUD as these mHealth offerings continue to grow. Telemedicine practices that have long provided virtual OUD treatment to patients and that are utilizing evidence-based performance measures are well-positioned to conduct longitudinal analyses assessing the patterns of substance use, overdose, and hospitalization in patients treated through telemedicine. Additionally, mixed methods research will be critical to assess the health equity impact of evidence-based telemedicine apps for OUD. Communities with lower levels of technological literacy or limited infrastructure for reliable internet or cellular connectivity may experience barriers to utilizing telemedicine apps for OUD treatment, limiting the potential health benefits to these populations. Simultaneously, expanded telemedicine services for OUD could improve medication access for populations such as Black, Hispanic, Asian, and Native American communities that face lower access to buprenorphine treatment compared to White persons [6]. Qualitative and quantitative implementation science research will be important to assess the usability and acceptability of telemedicine apps for these communities and identify appropriate implementation strategies to facilitate care access.

Finally, in the future, researchers could also consider telemedicine apps as platforms for conducting addiction research just as office-based OUD programs function as research settings. App-based virtual models of OUD care can be used to test innovations such as pharmacy partnerships for buprenorphine delivery, [7] contingency management programs, [8] and integration of novel therapies such as glucagon-like peptide 1 agonists (GLP-1s) [9]. As GLP-1s show potential to address unmet needs for polysubstance use treatment, [10] telemedicine programs for OUD treatment could act as clinical trial sites relying on their app platforms to study participants and collect real-time data on health outcomes. In the next few years, evidence-based smartphone apps could form the foundation of a broad telemedicine research network to advance the treatment of OUD.

## Data Availability

No datasets were generated or analysed during the current study.

## Abbreviations

OUD: Opioid use disorder
GLP-1: Glucagon–like peptide 1 agonists

## References

[1] Williamson A, Heydarshahi B, Finley-Abboud D, et al. What smartphone apps exist to support recovery from opioid use disorder? A content analysis of publicly available opioid-related smartphone apps. Addict Sci Clin Pract. 2025;20(1):26. 10.1186/s13722-025-00549-y. 40075426 10.1186/s13722-025-00549-y PMC11905484

[2] Lira MC, Jimes C, Coffey MJ. Retention in telehealth treatment for opioid use disorder among rural populations: A retrospective cohort study. Telemed J E Health. 2023;29(12):1890–6. 10.1089/tmj.2023.0044. 37184856 10.1089/tmj.2023.0044 PMC10714254

[3] Chan B, Cook R, Levander X, et al. Buprenorphine discontinuation in telehealth-only treatment for opioid use disorder: A longitudinal cohort analysis. J Subst Use Addict Treat. 2024;167:209511. 10.1016/j.josat.2024.209511. 39243979 10.1016/j.josat.2024.209511 PMC12002408

[4] Burke B, Clear B, Rollston RL, Miller EN, Weiner SG. An assessment of the One-Month effectiveness of telehealth treatment for opioid use disorder using the brief addiction monitor. Subst Use Addctn J. 2024;45(1):16–23. 10.1177/29767342231212790. 38258856 10.1177/29767342231212790

[5] Coffey MJ, Weng M, Jimes C, Brigham S, Lira MC. Telehealth treatment for opioid use disorder during pregnancy. JAMA Netw Open. 2024;7(3):e242463. 10.1001/jamanetworkopen.2024.2463. 38483393 10.1001/jamanetworkopen.2024.2463 PMC10940952

[6] Nedjat S, Wang Y, Eshtiaghi K, Fleming M. Is there a disparity in medications for opioid use disorder based on race/ethnicity and gender? A systematic review and meta-analysis. Res Social Adm Pharm. 2024;20(3):236–45. 10.1016/j.sapharm.2023.12.001. 38101952 10.1016/j.sapharm.2023.12.001

[7] Lira MC, Hendy LE, Liakas A, et al. Early findings on home delivery of buprenorphine and retention in treatment for opioid use disorder. Addict Sci Clin Pract. 2025;20:14. 10.1186/s13722-025-00545-2. 39939871 10.1186/s13722-025-00545-2 PMC11816807

[8] Bolívar HA, Klemperer EM, Coleman SRM, DeSarno M, Skelly JM, Higgins ST. Contingency management for patients receiving medication for opioid use disorder: A systematic review and Meta-analysis. JAMA Psychiatry. 2021;78(10):1092–102. 10.1001/jamapsychiatry.2021.1969. 34347030 10.1001/jamapsychiatry.2021.1969 PMC8340014

[9] Volkow ND, Xu R. GLP-1R agonist medications for addiction treatment. Addiction. 2025;120(2):198–200. 10.1111/add.16626. 39049203 10.1111/add.16626 PMC12771407

[10] Lira MC, Barrett E, Coffey MJ. GLP-1 receptor agonists: encouraging signals for treating alcohol use disorder. J Gen Intern Med. 2025;40:2997–2999. 10.1007/s11606-025-09498-3 10.1007/s11606-025-09498-3 PMC12463799 40272681

